# Computed Tomography (CT) Scanning Facilitates Early Identification of Neonatal Cystic Fibrosis Piglets

**DOI:** 10.1371/journal.pone.0143459

**Published:** 2015-11-23

**Authors:** Antoine Guillon, Claire Chevaleyre, Celine Barc, Mustapha Berri, Hans Adriaensen, François Lecompte, Thierry Villemagne, Jérémy Pezant, Rémi Delaunay, Joseph Moënne-Loccoz, Patricia Berthon, Andrea Bähr, Eckhard Wolf, Nikolai Klymiuk, Sylvie Attucci, Reuben Ramphal, Pierre Sarradin, Dominique Buzoni-Gatel, Mustapha Si-Tahar, Ignacio Caballero

**Affiliations:** 1 INSERM, Centre d’Etude des Pathologies Respiratoires, UMR 1100/EA6305, Tours, France; 2 CHU Tours, service de réanimation polyvalente, Tours, France; 3 INRA, UMR1282 ISP, Nouzilly, France; 4 INRA, UMR1277 PFIE, Nouzilly, France; 5 INRA, CIRE, Nouzilly, France; 6 CHU Clocheville, Tours, France; 7 CHU Tours, service anesthésie-réanimation, Tours, France; 8 Gene Center and Center for Innovative Medical Models (CiMM), LMU Munich, Germany; Lee Kong Chian School of Medicine, SINGAPORE

## Abstract

**Background:**

Cystic Fibrosis (CF) is the most prevalent autosomal recessive disease in the Caucasian population. A cystic fibrosis transmembrane conductance regulator knockout (CFTR-/-) pig that displays most of the features of the human CF disease has been recently developed. However, *CFTR* ^-/-^ pigs presents a 100% prevalence of meconium ileus that leads to death in the first hours after birth, requiring a rapid diagnosis and surgical intervention to relieve intestinal obstruction. Identification of *CFTR* ^-/-^ piglets is usually performed by PCR genotyping, a procedure that lasts between 4 to 6 h. Here, we aimed to develop a procedure for rapid identification of *CFTR* ^-/-^ piglets that will allow placing them under intensive care soon after birth and immediately proceeding with the surgical correction.

**Methods and Principal Findings:**

Male and female *CFTR* ^+/-^ pigs were crossed and the progeny was examined by computed tomography (CT) scan to detect the presence of meconium ileus and facilitate a rapid post-natal surgical intervention. Genotype was confirmed by PCR. CT scan presented a 94.4% sensitivity to diagnose *CFTR* ^-/-^ piglets. Diagnosis by CT scan reduced the birth-to-surgery time from a minimum of 10 h down to a minimum of 2.5 h and increased the survival of *CFTR* ^-/-^ piglets to a maximum of 13 days post-surgery as opposed to just 66 h after later surgery.

**Conclusion:**

CT scan imaging of meconium ileus is an accurate method for rapid identification of *CFTR* ^-/-^ piglets. Early CT detection of meconium ileus may help to extend the lifespan of *CFTR* ^-/-^ piglets and, thus, improve experimental research on CF, still an incurable disease.

## Introduction

Cystic fibrosis (CF) is a recessive genetic disorder caused by mutations in the cystic fibrosis transmembrane conductance regulator (*CFTR*) gene, rendering the protein non-functional [1]. CFTR has been shown to function as a regulated chloride and hydrogen carbonate channel at the cell surface. Mutations in the *CFTR* gene affect the rheology of secretions, which become thick and difficult to clear from respiratory airways [2]. This chronic obstruction of the airways caused by viscous secretions is the most challenging problem in the treatment of CF-related pulmonary disorders. Chronic CF is characterized by the lung’s inability to clear the tissue of opportunistic pathogens, such as *Pseudomonas aeruginosa*. This is probably due to several factors including impairment of the innate immune system, the exacerbated recruitment of polymorphonuclear neutrophils (PMNs) into the lungs, excessive release of proteases and ultimately chronic lung obstruction [1, 3, 4]. The latter is the main cause of death in CF patients.

Although several transgenic mouse strains have been generated in order to study the pathophysiology of CF, their usefulness have been limited by the lack of a phenotype in the respiratory tract that mimics the complications observed in human CF [5, 6]. More recently, this drawback has been tackled by the development of a mutated CFTR^∆F508/∆F508^ pig [7] and a CFTR^-/-^ knockout pig [8, 9] (hereafter called CF pigs). The latter CF pig model develops both intestinal (meconium ileus, microcolon) and lung pathology similar to the alterations described in CF patients [10, 11]. This feature makes the CF pig a very valuable model to study the pathogenesis of CF and to evaluate new therapies. However, the incidence of meconium ileus (MI) at birth in the CF pigs is 100% compared to 13–17% in humans [12]. The severity of the MI makes this process lethal unless ileostomy is performed early to bypass the obstruction. Despite the surgical correction of the obstruction, the survival rate of the piglets remains very low [13]. This problem hampers the availability of adult pigs as a model to study chronic infections in the adult host as they occur in case of the human disease.

Several factors could improve the outcome of the surgical procedure in CF piglets. Among those factors, the interval between birth and the performance of ileostomy could be of importance to improve piglets survival, since their health deteriorates quickly after birth [8, 9]. Rapid identification of the CF piglets is therefore necessary in order to proceed with the ileostomy. Identification of *CFTR* ^-/-^ piglets is usually performed by PCR screening of the locus of interest, a procedure that lasts between 4 to 6 h. A more rapid diagnosis could be made by taking advantage of the pathological alterations that are consistently found in CF pigs. Since a 100% prevalence of MI is expected in CF pigs, imaging techniques may be useful to diagnose the presence of MI, allowing the rapid screening of CF piglets. To date, obstructive bowel disorders such as MI can be detected by their ultrasound (US) and radiological features in humans [14, 15]. Abdominal US in human neonates can detect multiple loops of bowel filled with hyperechoic thick meconium [16]. However, meconium ileus is commonly missed in human; sonographic characteristics of fetal bowel obstruction are neither sensitive nor specific [17–19]. Radiographic findings include dilated bowel loops proximal to the obstruction, however they are also of limited value to differentiate MI from other causes of acute abdomen [20]. Computed tomography (CT) scan helps to improve the differential diagnosis with other diseases. Typical findings include abnormal dilation of bowel loops filled with homogenous material [20].

The aim of this study was to evaluate the usefulness of CT scan to identify neonatal CF piglets. The sensitivity and the specificity of the imaging assay was ultimately compared to PCR diagnosis. We observed that CT scan imaging was a rapid and accurate procedure to identify the *CFTR* ^-/-^ piglets. This procedure allowed decreasing the time from birth to placement of the piglets under intensive care and surgery, which may help to improve the survival of the *CFTR* ^-/-^ piglets.

## Methods

### Animals

All experiments were conducted in accordance with the guidelines of the Institutional Animal Care and Use Committee at INRA. The protocol was approved by the “Comité d'Ethique en Expérimentation Animale Val de Loire” (n° 00028.01). All surgery was performed under isoflurane anaesthesia, and all efforts were made to minimize post-operatory suffering. Those piglets showing severe distress and a deteriorating health status were sacrificed with an i.v. overdose of pentobarbital (Dolethal, Vétoquinol, France).

*CFTR* ^+/-^ pigs were produced by replacing the exon 1 of the *CFTR* gene by a STOP box and a neo cassette using homologous recombination by BAC vectors [9]. Single male and female *CFTR* ^+/-^ transgenic pigs were moved to INRA, Nouzilly (France) and mated to generate *CFTR* ^+/+^, *CFTR* ^+/-^ and *CFTR* ^-/-^ piglets. A total of 102 piglets from 6 different litters were used in the experiments. Newborn piglets were allowed to suckle colostrum for 1 h before being subjected to CT scan imaging. Genotype of the piglets was confirmed by PCR. Within 3–12 h after birth, control and CF piglets underwent surgery for an ileostomy to prevent complications from MI as described below. Procedures applied to the piglets from birth to the surgery are summarized in Table 1.

**Table 1:** Mean time and procedures applied to newborn piglets subjected or not to CT scan diagnosis.

	Farrowing	Suckling	Genotyping/CT scan	Surgical preparation	Ileostomy
**Without CT scan**	3–4 h	1 h	4–6 h	1.5–2.5 h	1 h
**With CT scan**	*	1 h	5–10 min**	1.5–2.5 h	1 h

* Newborn piglets were diagnosed by CT scan after 1 h suckling without waiting for the end of farrowing.

** Time per piglet: individual preparation for surgery started immediately after scanning.

### Diagnosis of CFTR^-/-^piglets

Diagnosis of those piglets carrying a homozygous deletion of the *CFTR* gene and thus MI was evaluated by abdominal imaging (CT scan). The genotype of newborn animals subjected to CT scan imaging was later confirmed by PCR.

**Fig 1:**
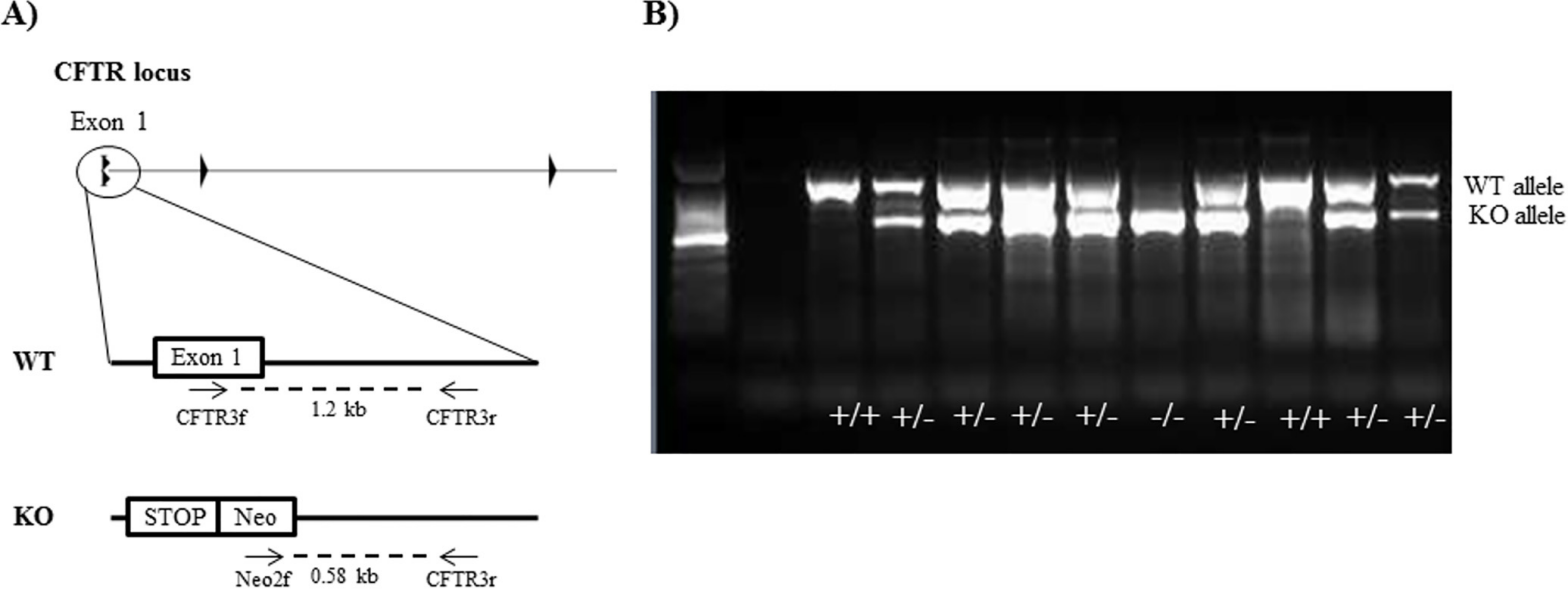
Genotyping of newborn transgenic piglets by PCR. A) Schematic representation of the PCR genotyping strategy. A multiplex PCR was setup with a combination of 3 different primers (arrows) binding to exon 1, intron 1 or the neo cassette that allowed to screen the loss of the wild-type (WT) allele. B) Agarose gel showing genotyping by PCR of *CFTR* ^+/+^ (single band at 1.2 kb), *CFTR* ^+/-^ (double band at 1.2 and 0.58 kb) and *CFTR* ^-/-^ (single band at 0.58kb) piglets.

Briefly, the genotype of the *CFTR* ^+/+^, *CFTR* ^+/-^ and *CFTR* ^-/-^ piglets was determined by multiplex PCR using a combination of 3 different primers. The primers used were as follows: CFTR3f: 5-gacagtactgcttagtggtcag-3, CFTR3r: 5-cagatctagaattctggtatg-3, Neo2f: 5-gagatgaggaagaggagaacag-3. Primer pair CFTR3f/CFTR3r covered a 1.2-kb sequence between exon 1 and intron 1 of the *CFTR* gene, while primer pair Neo2f/CFTRr covered a 580-bp sequence from the neo®/kan® cassette to intron 1 (Fig 1). Primer concentrations in the reaction mix were 0.49 μM for each primer set. Annealing temperature was 59°C.

CT scan imaging was performed on 93 newborn piglets under anaesthesia with isoflurane at a concentration of 4%. Anesthetized animals had a normal and spontaneous respiration. They were placed in prone position and imaging of the abdomen was carried out using a Siemens Somatom Definition AS^®^ 128. The acquisition parameters were set to 200 mA and 120 kV. Slices were 0.6 mm thick and acquisition time was 6 seconds. The CT images ranged from -1000 to 1000 on the Hounsfield Unit (HU) scale. In order to identify the *CFTR* ^-/-^ piglets, the CT images were set to a window width of 300 HU and contrast window of 40 HU. The filter for reconstruction was I-40. Piglets were diagnosed as positive when a dilation of the bowel loops filled with dense material was observed.

### Ileostomy and post-surgical care

Ileostomy was performed on 22 piglets (weight 1–1.5 kg) as soon as they were diagnosed as *CFTR* ^-/-^. Thirteen of those piglets were rapidly diagnosed by CT scan, while 9 piglets were subjected to surgery after PCR genotyping. Three wild-type (WT) piglets were also subjected to ileostomy as a control to evaluate the safety of the surgical procedure.

Animals were anesthetized with isoflurane in oxygen, orotracheally intubated and mechanically ventilated. A 24-gauge intravenous catheter was placed in the brachial vein and a sodium-potassium-glucose solution mixture was infused continuously. Atracurium was administered for muscle relaxation. Post-operative pain was controlled with finadyne (2 mg/kg), buprenorphine (100 μg every 8h) and paracetamol (10 mg/kg every 6h). Prophylactic antibiotics were administered: ampicillin (10 mg/kg every 8h), metronidazole (22 mg/kg every 8h) and gentamicin (3 mg/kg per day). Then, piglets were fed with colostrum or milk replacements and received: i) oral administration of pancreatic enzymes (5000 UI of lipase per day), ii) oral fat-soluble vitamins, oral proton pump inhibitor (1 mg/kg per day) (Eupantol®) and iii) polyethylene glycol 3350 (Colopeg®) with each meal to maintain soft stools. The health status of the piglets was supervised by a veterinarian.

### Histopathological evaluation of CFTR^-/-^ piglet intestines

At necropsy, piglets were examined for macroscopic lesions. Tissues were collected and fixed in 4% formalin for at least 1 week and paraffin-embedded. Five-μm tissue sections were routinely stained with haematoxylin and eosin (H&E) for histological examination.

### Statistical analysis

Sensitivity, specificity, positive and negative predictive values of the different diagnostic tests were analysed using MedCalc for Windows, version 13.3.1 (MedCalc Software, Ostend, Belgium). Box and whisker plot analysis were used to display the variation and median of the birth-to-surgery time. The boxes show the 25th, 50th (median) and 75th percentiles, and whiskers show the minimum and maximum times that are not outliers or extreme values. Post hoc pairwise comparisons were conducted using the Mann-Whitney U-test. Survival curves of *CFTR* ^-/-^ piglets between piglets diagnosed or not by CT scan were compared using chi square analysis (GraphPad Prism software; San Diego, CA).

## Results

### CT scan imaging is an accurate method for diagnosis of MI in CFTR^-/-^ newborn piglets

**Fig 2:**
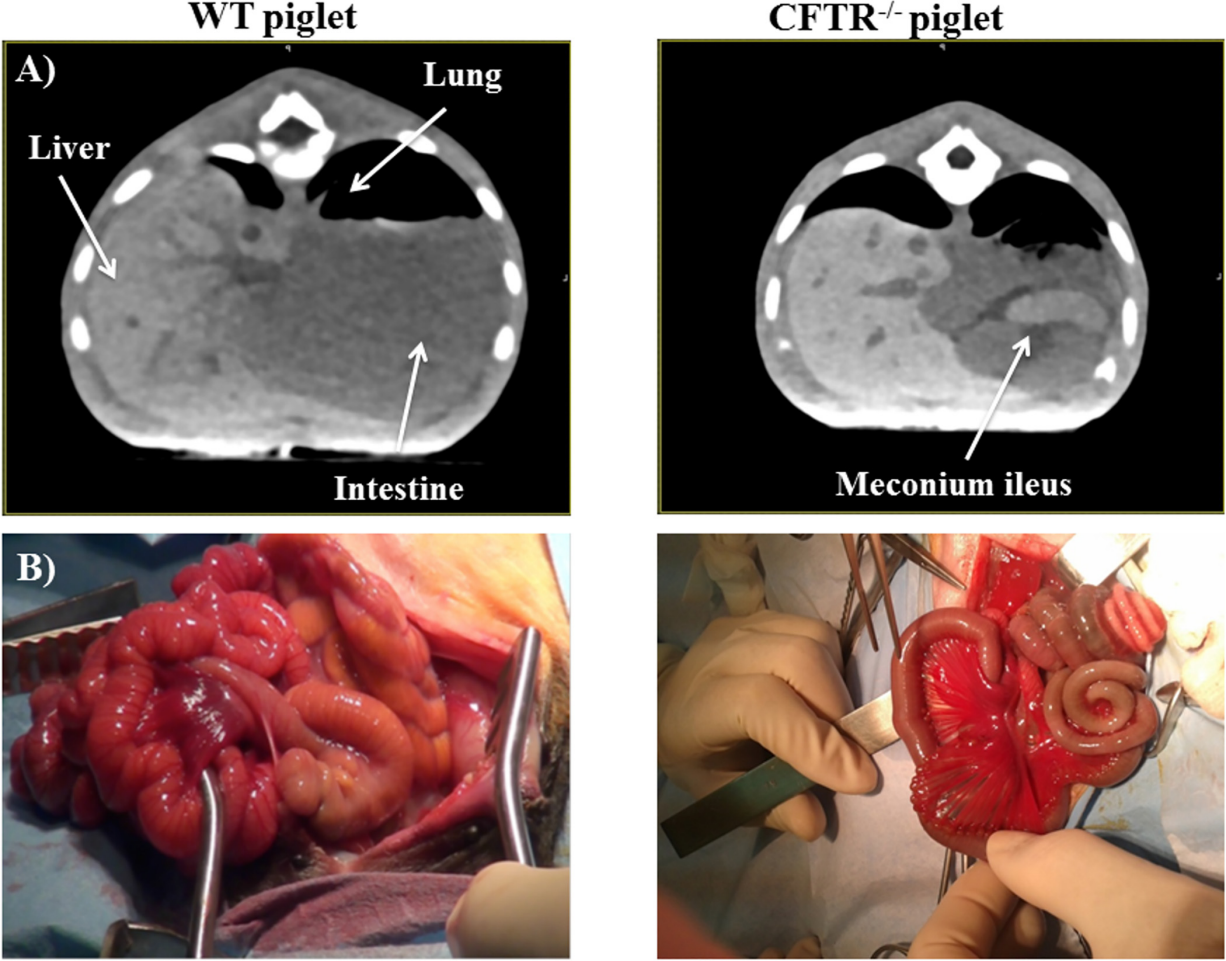
Meconium ileus in the *CFTR* ^-/-^ newborn piglet. A) CT scan images of WT and *CFTR* ^-/-^ abdomen. The presence of MI is observed in *CFTR* ^-/-^ as a homogenous dense material filling the intestinal loops of the piglets. B) Macroscopic image of WT and *CFTR* ^-/-^ intestines. The presence of MI and microcolon can be observed in the *CFTR* ^-/-^ piglet.

A total of 93 newborn piglets were subjected to CT scan. CT scan imaging displayed the presence of MI as a very dense homogenous content in the intestines. There was a clear increase of 20 to 30 HU units in the MI of CFTR^-/-^ piglets. These enhanced HU eased the diagnosis of the CFTR^-/-^ piglets (Fig 2).

When the results obtained by CT scan were compared to those obtained by PCR genotyping, we observed that 84 piglets were accurately diagnosed as CF and non-CF piglets, with only 1 false negative and 8 false positive results out of 93 piglets analysed. Indeed, the prediction of CT scan to diagnose CFTR-/- newborn piglets had a sensitivity of 94 (73–99) %, a specificity of 89 (80–95) %, a positive predictive value of 68 (47–85) % and a negative predictive value of 99 (92–100) %. As such, CT scan imaging proved to be a very sensitive and specific diagnostic method for the screening of CFTR^-/-^ piglets (Table 2).

**Table 2:** CT scan imaging sensitivity, specificity, positive predictive value (PPV) and negative predictive value (NPV) to diagnose CFTR^-/-^ newborn piglets.

	Number of animals	Number of *CFTR* ^-/-^	Sensitivity	Specificity	PPV	NPV
**CT scan**	93	18	94.4% (72.63–99.07)	89.33% (80.05–95.27)	68% (46.50–85.01)	98.53% (92.05–99.75)

^95% confidence interval is indicated between brackets.^

### CT scan imaging significantly decreases the birth-to-surgery time of CFTR^-/-^ piglets

**Fig 3:**
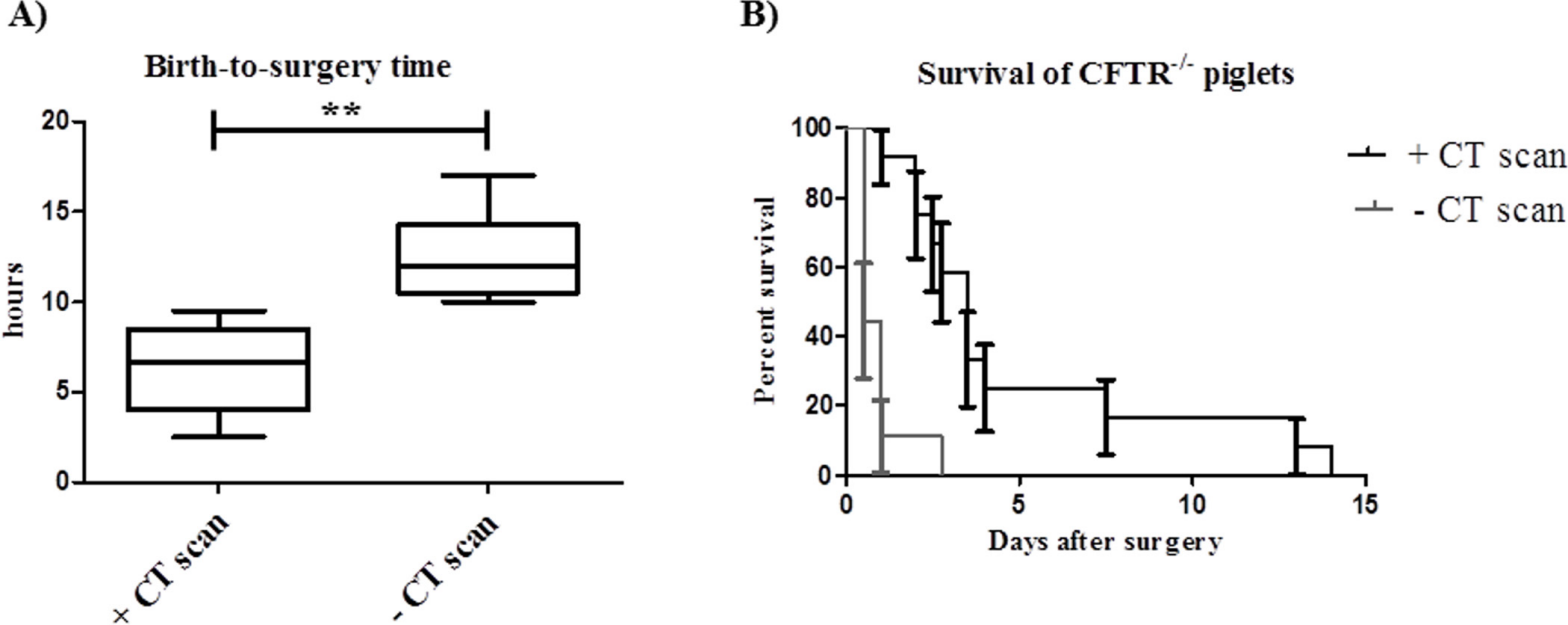
The use of CT scan decreases the birth-to-surgery time and increases the survival rate of *CFTR* ^-/-^ piglets. A) Box and whiskers plot of the birth-to-surgery time of *CFTR* ^-/-^ piglets diagnosed or not by CT scan (** indicates p<0.001). B) Survival curve of *CFTR* ^-/-^ piglets diagnosed or not by CT scan.

In contrast to CT scan which allowed us to proceed with the diagnosis as soon as the first piglet had the colostrum (i.e. 1 h after birth of the first animal), in the case of genotyping all samples were processed at once (i.e. after birth of the last animal). Taking into account a farrowing time of at least 3–4 hours, this further increased the birth-to-surgery time for genotyped animals. Besides, the duration of the diagnosis by CT scan ranged between 5 to 10 min per piglet. Ultimately, this led to a significant decrease in the birth-to-surgery time from a minimum of 10 h down to a minimum of 2.5 h. The median time from birth to the surgical intervention was significantly reduced from 12 to 6.7 h (p<0.001). This diagnostic method allowed performing an ileostomy in all the CFTR^-/-^ piglets from the same litter (2 to 6 piglets per litter) in less than 10 h after birth as opposed to a maximum of 17 h in the piglets diagnosed by genotyping (Fig 3A).

Although the mortality rate after the ileostomy procedure is still very high in the *CFTR* ^-/-^ piglets, we were able to increase the survival time up to 13 days in animals that were rapidly diagnosed compared to 2.5 days when the surgical correction of the intestinal obstruction was delayed for longer than 10 h (Fig 3B). The *CFTR* ^+/+^ piglets that underwent an ileostomy survived longer than 1 month and did not present any significant health problems (data not shown).

**Fig 4:**
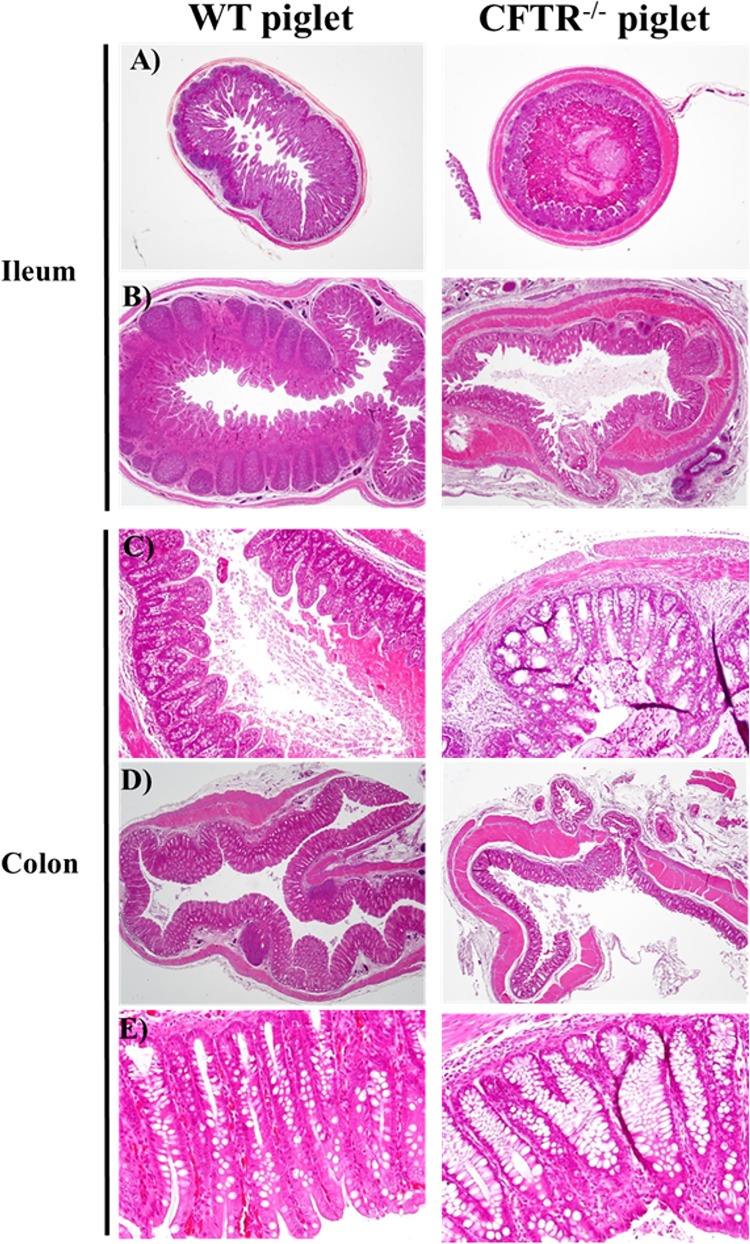
Intestinal phenotype of newborn and 13 days old *CFTR* ^-/-^ piglets. H&E staining of WT and *CFTR* ^-/-^ ileum and colon at day 1 [A (x 20) and C (x 100)] and day 13 [B (x 20), D (x 20) and E (x 200)] after birth. *CFTR* ^-/-^ piglets showed a hypertrophy of mucus cells with mucus accumulation, thickening of the muscular wall and presence of diverticuli. A significant atrophy of the lymphoid follicles in the mucosal lamina propria of the ileum can be observed at day 13 after birth.

Histopathological analysis of the ileum and colon of newborn *CFTR* ^-/-^ piglets showed a pathological phenotype in agreement with previous studies [9, 21]. Briefly, *CFTR* ^-/-^ showed an atrophy of the intestinal mucosa with a hypertrophy of the mucus cells and accumulation of mucus in the lumen compared to WT animals. The muscular wall of the ileum and colon was thickened and more fragile in the *CFTR* ^-/-^ piglets with the presence of diverticulosis. Similar changes were observed in 13 days old *CFTR* ^-/-^ piglets (n = 2). In addition, it is important to notice the lack of development of the lymphoid follicles in the mucosal lamina propria of the ileum (Fig 4).

## Discussion

Since the discovery and cloning of the *CFTR* gene, a wide range of mouse models have been developed to study the disease [22]. Differences between mice and humans in lungs size, airway architecture and development of the typical CF lung disease render them of limited significance [6, 23]. In that regard, the generation of the recent CF pig model seemed to overcome the problems found in the mouse model. Porcine lungs share many anatomical, histological, biochemical, and physiological features with human lungs [3] and more important, *CFTR* ^-/-^ develop hallmark features of CF lung disease [11]. In addition, the inflammatory response to *Pseudomonas aeruginosa*, the major pathogen in CF lung disease, in pigs presents striking similarities to humans [4].

However, all the benefits of the pig model are hampered due to the high occurrence of a severe intestinal obstruction by MI, which leads to early death [8]. Besides, surgical correction of the obstruction by an ileostomy results in high mortality rates [13]. We have observed that newborn *CFTR* ^-/-^ piglets have a hypertrophic muscular wall in the ileum with the presence of numerous diverticuli. The pathogenesis of diverticulosis is likely the result of pressure increase by the meconium that leads to herniation of the mucosa [21]. This situation could be exacerbated in the first hours after birth due to the food ingestion by the piglets leading to intestine perforation and peritonitis. Performing an ileostomy in these piglets is thus an emergency to decrease the occurrence of fatal intestine perforation. Here, we attempted to reduce the birth-to-surgery time of the newborn *CFTR* ^-/-^ piglets by using CT scan imaging as diagnostic tool.

CT scan imaging is a very sensitive and specific tool to detect the presence of MI in the intestine. CT scan images of MI showed a very distinct dense and homogenous mass that filled enlarged bowel loops in accordance with the descriptions found in the literature [14, 24]. This tool allowed us to quickly sort those pigs carrying the homozygous mutation and perform a surgical correction in the intestinal obstruction.

During our study, we have managed to keep alive newborn piglets diagnosed by CT scan for 13 days, an improvement when compared to a maximum of 2.5 days before early diagnosis of the CF piglets. Our results suggest that a better survival rate may be obtained in those piglets that were quickly diagnosed and subjected to an ileostomy. Newborn piglets are very vulnerable animals, poorly equipped to keep body heat. To provide the energy required they have to maximise the colostrum intake. However, CF piglets present with severe MI, intestinal obstruction and distension. In this case, it was important to decrease the amount of food intake in order not to aggravate the intestinal obstruction that could lead to intestinal perforation and peritonitis. Although a limit of 1 h suckling colostrum was set for all the newborn piglets, those that did not undergo placement of the ileostomy early after birth (<8 h) had to be re-fed in order to obtain the energy needed. On the other hand, early detection of CF piglets allowed us to immediately place them under intensive care, including fluidotherapy. This system allowed limiting the colostrum intake to 1 h while keeping a good energetic balance, which could be beneficial for the recovery of the piglets after surgery.

A careful interpretation of the data needs to be taken into account. CT scan diagnosis was implemented later in the course of the studies and greater experience in the surgical procedure and post-operative care may be related to the observed increase in the survival rates of the CF piglets. However, we believe this is not the case. The surgical procedure was first established in the *CFTR* ^*+/+*^ piglets, which survived longer than 1 month, and no improvement in the recovery or physiological state was observed in WT piglets that were subjected to an ileostomy later during the course of the studies. Moreover, 3 out of 9 *CFTR* ^*-/-*^ piglets that were not subjected to CT scan diagnosis and early surgery had already started to develop early signs of intestinal perforation and peritonitis, probably due to excessive pressure in the intestines by the meconium ileus and food ingestion.

Despite early treatment of the *CFTR* ^*-/-*^ piglets, the survival rate was less than that obtained by other laboratories using piglets with different genetic background. Previous studies have reported a survival of 2 months or longer in piglets that underwent ileostomy [13]. These differences in the survival rate and the intestinal disease severity may be related to the genetic background of the piglets. In fact, different genetic predisposition to diverticulosis has been proposed for different pig breeds [25]. Further advances to improve CF pig lifespan include the development of a *CFTR* ^-/-^ model, where transgenic expression of the *CFTR* gene is under control of the intestinal fatty acid-binding protein (*FABP2*) promoter. The *FABP2* gene is mainly expressed in the intestinal tract [26]. Thus, the *FABP2* promoter could be used to induce a localized expression of the CFTR gene in the intestines. This strategy was first used in a CF mouse model by Zhou and colleagues using rat *Fabp2* to drive the expression of human CFTR in mice [27]. A similar strategy was employed by Stoltz and colleagues in the CF pig model. They generated a CFTR^-/-^ pig where the expression of wild-type CFTR was driven by the rat *Fabp2* promoter (called CFTR^-/-^;TgFABP>pCFTR pigs) [28]. This new transgenic model was able to partially restore CFTR expression and function in the intestines, alleviating the appearance of MI. CFTR expression was shown to be restricted to the intestines and CFTR^-/-^;TgFABP>pCFTR pigs were able to survive longer than 9 months developing spontaneous lung disease [28]. However, these animals still require intensive post-natal care and its widespread international use is likely to be hampered due to sanitary restrictions.

Nevertheless it is important to highlight the importance of animal models with different genetic backgrounds in order to better understand CF pathogenesis. In this regard, CF is a complex disease characterized by substantial clinical heterogeneity [29]. This variability may be related with the expression of different “modifier” genes expressed by different genetic backgrounds, which can affect the severity of the observed phenotype [22]. The CF pig model produced in this study seems to develop a very severe form of the intestinal phenotype. We have observed some abnormalities not described yet such as the lower development of the gut-associated lymphoid tissue (GALT) in the ileum. To the best of our knowledge, it is the first time that under-developed GALT was reported in a CF context. On the contrary, it is well known that lack of enteral nutrition reduces GALT in human and is associated with higher postoperative infectious complication rates [30, 31]. Herein, pre- and postoperative nutritional support strategies were identical for WT or CFTR^-/-^ piglets and are thus unlikely to be responsible. However, amniotic fluid was unable to pass through the digestive system of CFTR^-/-^ piglets in utero, due to the intestinal obstruction (as illustrated by the under-developed colon in CFTR^-/-^ piglets). Amniotic fluid is now considered as a complex and dynamic milieu for the foetus with important nutritive and protective functions [32]. Therefore, we suspect that the intestinal obstruction of CF piglets also avoid the absorption of nutrient from the amniotic fluid circulation cycle during the foetal development [33]. It could be an important factor that impairs the development of GALT in CFTR^-/-^ piglets. These differences between genetic backgrounds should be regarded as an advantage of the model, since they may provide the basis for later studies on “modifier” genes that may play a role in the severity of the disease.

In conclusion, CT scan imaging is a reliable method for screening *CFTR* ^-/-^ piglets. A quick diagnosis and surgical placement of an ileostomy may help to improve the survival expectancy of the *CFTR* ^-/-^ piglets. Although further efforts are needed to improve the survival of CF pigs, this model can be already of use for the study of the early development of CF.
